# The reliability, correlation with clinical symptoms and surgical outcomes of dural sac cross-sectional area, nerve root sedimentation sign and morphological grade for lumbar spinal stenosis

**DOI:** 10.1186/s12891-023-06353-6

**Published:** 2023-03-25

**Authors:** Jin Yang, Yiling Xiong, Yuexuan Hu, Mei Huang, Li Zhang, Xia Pu, Qiuhan Li

**Affiliations:** 1 Department of Orthopedic Surgery, Affiliated Hospital of Southwest Medical University, 25 Taiping Road, Luzhou, Sichuan 646000 China; 2 Department of Radiology, Affiliated Hospital of Southwest Medical University, 25 Taiping Road, Luzhou, Sichuan 646000 China; 3 Department of Clinical Skills Center, Affiliated Hospital of Southwest Medical University, 25 Taiping Road, Luzhou, Sichuan 646000 China

**Keywords:** Lumbar spinal stenosis, Dural sac cross-sectional area, Morphological grade, Nerve root sedimentation sign, Reliability, Correlation, Symptoms

## Abstract

**Background:**

No study had directly compared the reliability, correlation with clinical symptoms, and surgical outcomes of dural sac cross-sectional area (DCSA), nerve root sedimentation sign (SedSign), and morphological grade for lumbar spinal stenosis (LSS).

**Methods:**

From January 2017 to December 2020, 202 patients with LSS were retrospectively analyzed. The narrowest segments were assessed via T2-weighted cross-sectional images using DCSA, morphological grade, and SedSign by two independent observers. Three classifications’ reliabilities were evaluated. Correlations between three classifications and between each of the classifications and symptoms or surgical outcomes 12 months postoperatively were evaluated.

**Results:**

There were 144 males and 58 females; 23, 52, and 127 patients had the narrowest segment in L2–3, L3–4, and L4–5, respectively. The intra-observer reliability of DCSA ranged from 0.91 to 0.93, and the inter-observer reliability was 0.90. The intra-observer reliability of SedSign ranged from 0.83 to 0.85, and the inter-observer reliability was 0.75. The intra-observer reliability of morphological grade ranged from 0.72 to 0.78, and the inter-observer reliability was 0.61. Each of these classifications was correlated with the other two (*P* < 0.01). For preoperative symptoms, DCSA was correlated with leg pain (LP) (*r* =  − 0.14), Oswestry Disability Index (ODI) (*r* =  − 0.17), and claudication (*r* =  − 0.19). Morphological grade was correlated with LP (*r* = 0.19) and claudication (*r* = 0.27). SedSign was correlated with ODI (*r* = 0.23). For postoperative outcomes, morphological grade was correlated with LP (*r* =  − 0.14), and SedSign was correlated with ODI (*r* = 0.17).

**Conclusions:**

Substantial to almost perfect intra and inter-observer reliabilities for the three classifications were found; however, these classifications had either weak correlations with symptoms and surgical outcomes or none at all. Based on our findings, using one of them without conducting other tests for LSS will have limited or uncertain value in surgical decision-making or evaluating the prognostic value.

## Introduction

Lumbar spinal stenosis (LSS) is defined as a decrease in the anatomical diameter or volume of the spinal canal and the compression of nerves and blood vessels due to bone or soft tissue hyperplasia and hypertrophy [1]. These changes are mainly revealed by medical imaging findings. However, the clinical manifestations of LSS demonstrate significant heterogeneity, including low back pain (LBP), leg pain (LP), lower limb paresthesia, and intermittent claudication; these symptoms are alleviated or disappeared during a state of rest. Moreover, during neurological examination, patients with LSS are rarely found with neurological deficits. Therefore, it is significant to effectively correlate symptoms with medical imaging findings to make a precise diagnosis and aid medical decision-making.

To better guide the treatment of LSS, several imaging classifications were proposed to evaluate LSS severity and attempt to correlate with preoperative symptoms and surgical outcomes of LSS [2–12]. Some widely accepted classifications mainly include quantitative methods, including the anterior and posterior diameter of the spinal canal and dural sac cross-sectional area (DCSA) [2–4], and qualitative ones, including the morphological grade of LSS severity proposed by Schizas et al. [6], nerve root sedimentation sign (SedSign) proposed by Barz et al. [7], and its modified version [8]. However, it has been reported that the sensitivity, specificity, and reliability of these classifications are different, and their correlations to clinical symptoms are debatable [4, 6–12]. Therefore, no consistently used or recommended imaging evaluation method is available [1, 13].

To the best of our knowledge, there is no direct comparison in DCSA, morphological grade, and SedSign for evaluating LSS. Therefore, this study aimed to analyze the reliability of these three classifications and their correlations with preoperative symptoms and clinical outcomes at 12 months postoperatively, determining which method can better aid clinical practice.

## Materials and methods

**Fig. 1:**
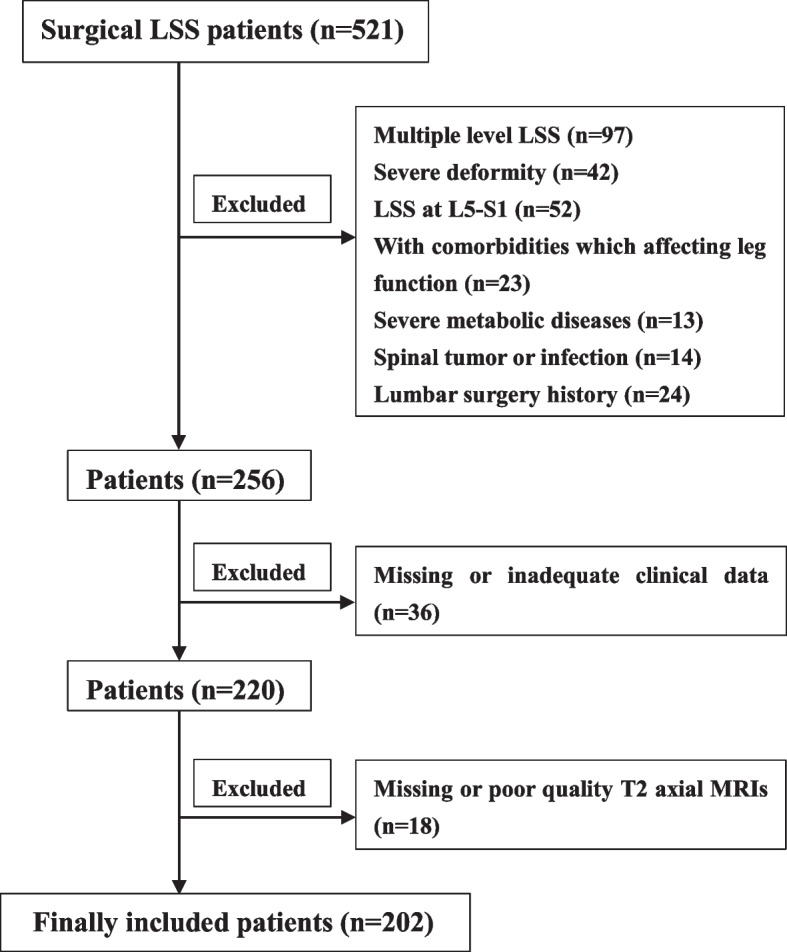
The flow chart of the included patients

This study retrospectively analyzed the data of 521 patients with LSS who underwent surgery in our hospital from January 2017 to December 2020. Following the inclusion and exclusion criteria, 202 patients were finally included in this study (Fig. 1). The following were the inclusion criteria: 1) patients with LSS (diagnosed based on their symptoms, signs, and imaging findings) who underwent surgery only at one level; 2) those with neurogenic intermittent claudication; 3) those with LP and with or without LBP; and 4) those with or without lower limb numbness, hyperalgesia, and other symptoms.

The exclusion criteria were as follow: 1) patients with the most stenotic level in L5/S1; 2) those with previous lumbar surgery history; 3) those with severe spinal deformity, such as scoliosis, kyphosis, and severe spondylolisthesis; 4) those with comorbidities affecting lower limb function, such as severe knee osteoarthritis or peripheral vascular diseases or neuropathy; 5) those with a spinal tumor or infection; 6) those with severe metabolic diseases, such as osteoporosis or thyroid hypofunction; and 7) those who had missing or poor quality of T2 axial magnetic resonance image (MRI) or inadequate clinical data.

### Measurement

#### Image observation and measurement

MRI scans (Philips Achieva, 1.5 Tesla) were performed on each patient in the supine position with both lower limbs extended. All evaluations were performed on cross-sectional T2-weighted images. For multi-segmental stenosis, the images of the narrowest segment (the surgical level) were selected for the following evaluation and analysis. Three images of the surgical level, including the most stenotic layer and above or below the layer, were collected and sent to two independent observers (a spinal surgeon and a neuroimaging diagnostic physician) who were blinded to all identifying information (including patients, treating clinicians, and clinical outcomes) and performed the measurements and observations based on the three classifications, including DCSA, morphological grade, and SedSign.

DCSA was measured and calculated using the method proposed by Hamanishi et al. [4]. We used the morphological grade with four degrees (A–D) proposed by Schizas et al. [6]. Grades A, B, C, and D indicated no stenosis or mild stenosis, moderate stenosis, severe stenosis, and extremely severe stenosis, respectively. We adopted the three subgroup classifications of SedSign proposed by Tomkins-Lane et al. [8]. “Negative” SedSign was defined as “all lumbar nerve roots located in the dorsal part of the dural sac, except the two ventral nerve roots exiting from the caudal to the level where the observations were being made.” “Positive” SedSign was defined as “the absence of nerve root sedimentation, with the majority of nerve roots located in the central aspect of the dura.” A “positive ( +)” SedSign indicated a positive SedSign with a room or empty space in the dura, and a “positive ( −)” SedSign indicated a positive SedSign without a room or empty space in the dura.

All observations and measurements were performed on the image browser workstation 4.1 (MedPacs, China). The measurement accuracy was ± 0.1 mm. The images of each segment were evaluated thrice at different time points. For qualitative methods (morphological grade or SedSign), to evaluate the correlation between the stenotic degree and symptoms, the final grade for the level was identified based on one of the following criteria: A) If evaluation results revealed the same grade ≥ 4 times, the grade was considered the final result; and B) If the evaluation results revealed the same grade < 4 times, the final decision regarding the grade was made by consensus between the two observers.

**Fig. 2:**
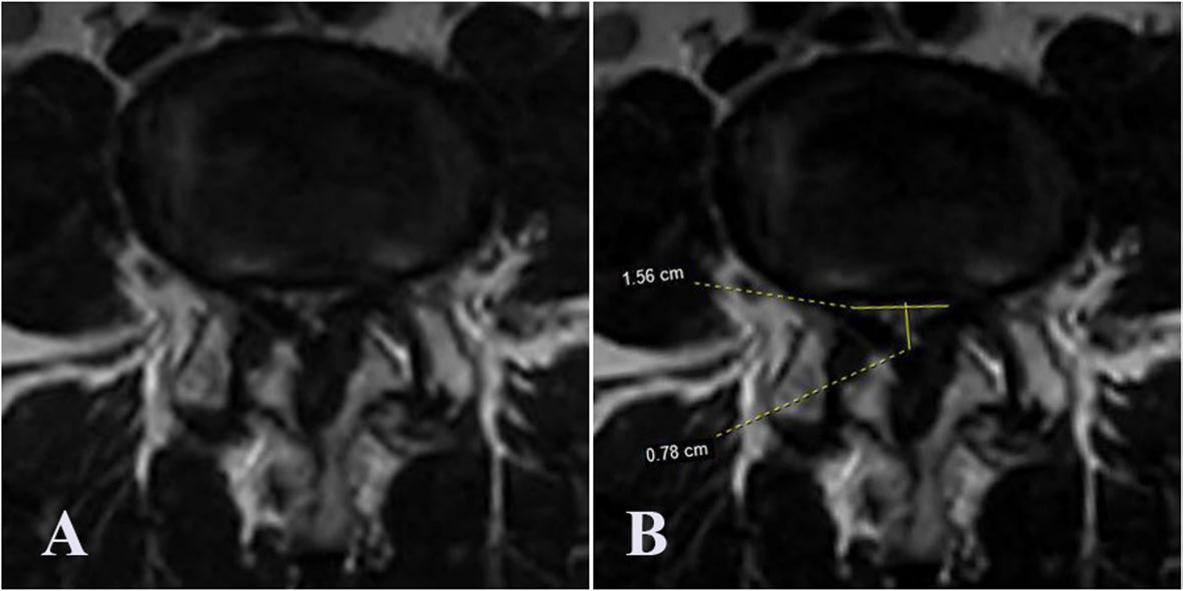
The image of the most stenotic layer of a 64-year-old man with L4–5 spinal stenosis. **A** the image is evaluated as morphological grade D and nerve root sedimentation sign (P [ −]); **B** the anterior–posterior and transverse diameter of the dural sac are 7.8 and 15.6 mm, respectively, and the dural sac cross-sectional area is 60.8 mm^2^ based on the calculation method of Hamanishi et al. [4]

A representative image is presented in Fig. 2.

### Clinical data

The symptomatic and functional parameters at the preoperative stage and 12 months postoperatively included a visual analog scale (VAS) for LBP and LP, the Oswestry Disability Index (ODI), and claudication distance. For patients with bilateral lower limb symptoms, the score in the leg with more severe symptoms was analyzed. The claudication distance based on patients’ reports was divided into the following four degrees: 1 =  > 500 m, 2 = 201–500 m, 3 = 50–200 m, and 4 =  < 50 m. Baseline data were obtained from the inpatient registry system, and follow-up data were acquired from outpatient or telephone visiting. This retrospective study was approved by the ethics committee of our hospital.

### Statistical analysis

SPSS version 24.0 (IBM Corp., Armonk, NY, USA) was used for statistical analysis, and the Shapiro–Wilk test was used to test the normality of all data. Continuous and normally distributed data were expressed as means ± standard deviations. Non-normal continuous data were represented by the median (interquartile range, IQR). An intraclass correlation coefficient test was conducted to detect the intra- and inter-observer reliability. The Spearman rank correlation coefficient was used to evaluate the correlations between each of the three imaging methods and the symptoms. *P* < 0.05 was considered statistically significant.

## Results

Of the 202 patients included, 144 (71.2%) were male and 58 (28.8%) were female. The median age was 69 (IQR, 62–76) years, and the median symptom duration was 48 (IQR, 16–81) months. The distributions of the narrowest segment were 23 (11.4%), 52 (25.7%), and 127 (62.9%) patients in L2–3, L3–4, and L4–5, respectively. Postoperatively, the symptoms of these patients were significantly improved (*P* < 0.01). The VAS of LBP decreased from 4.43 ± 1.16 preoperatively to 1.99 ± 1.11 postoperatively; VAS of LP from 6.60 ± 0.84 preoperatively to 1.49 ± 0.82 postoperatively; ODI from 44.66 ± 4.69 preoperatively to 21.35 ± 3.79 postoperatively; the claudication distance was also significantly improved (*P* < 0.01). More detailed information was presented in Table 1.

**Table 1:** Demographic Characteristics of 202 Patients

Characteristics	Median (Interquartile Range) or Mean ± Standard Deviation or n (%)
Age(years)	69 (62–76)
Sex male(%)	144 (71.2)
BMI	23 (23–26)
Duration of symptoms (months)	48 (16–81)
Smoker yes(%)	33 (16.3)
The types of lumbar spinal stenosis	
Central	9 (4.5)
Lateral recess	11 (5.4)
Foraminal	5 (2.5)
Central + Lateral recess	136 (67.3)
Central + Foraminal	41 (20.3)
The symptoms of lower extremities	
Unilateral	37 (18.3)
Bilateral	165 (81.7)
The most stenotic segment	
L2–3	23 (11.4)
L3–4	52 (25.7)
L4–5	127 (62.9)
Pre-operation	
VAS of low back pain	4.43 ± 1.16
VAS of leg pain	6.60 ± 0.84
ODI	44.66 ± 4.69
Claudication(m)	
> 500(%)	73 (36.1)
201–500(%)	25 (12.4)
50–200(%)	30 (14.9)
< 50(%)	74 (36.6)
12 months post-operatively	
VAS of low back pain	1.99 ± 1.11^a^
VAS of leg pain	1.49 ± 0.82^a^
ODI	21.35 ± 3.79^a^
Claudication(m) ^a^	
> 500(%)	139 (68.8)
201–500(%)	46 (22.8)
50–200(%)	16 (7.9)
< 50(%)	1 (0.5)

*VAS* Visual Analog Scale, *ODI* Oswestry Disability Index, *m* meter;

^a^ Statistical significance between the clinical symptoms of pre-operation and 12 months after surgery

**Fig. 3:**
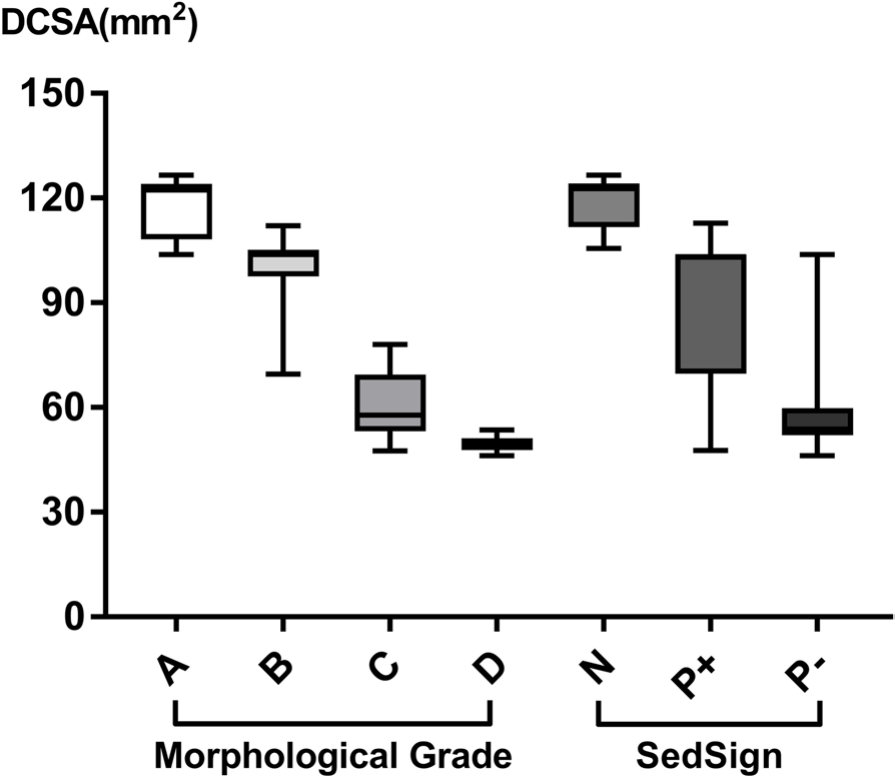
Average, minimum and maximum dural sac cross-sectional area (DCSA) for each degree of morphological grade and nerve root sedimentation sign (SedSign)

Based on the morphological grade, there were 11 (5.4%), 47 (23.3%), 113 (55.9%), and 31 (15.4%) patients with Grades A, B, C, and D, respectively. There were eight (3.9%) SedSign-negative patients, 68 (33.7%) SedSign-positive ( +) patients, and 126 (62.4%) SedSign-positive ( −) patients. The mean DCSA of all patients was 69.93 ± 22.21 mm^2^. The average, minimum, and maximum DCSAs for each degree of morphological grade and SedSign are presented in Fig. 3.

### Reliability

In these three classifications, the highest intra-observer reliability of the DCSA ranged from 0.91 to 0.93, and the inter-observer reliability was 0.90. The intra-observer reliability of the SedSign ranged from 0.83 to 0.85, and the inter-observer reliability was 0.75. The intra-observer reliability of the morphological grade ranged from 0.72 to 0.78, and the inter-observer reliability was 0.61 (Table 2).

**Table 2:** The Reliability of Dural Cross Sectional Area, Morphological Grade and Nerve Root Sedimentation Sign for lumbar spinal stenosis

	Observer	Intra-reliability(ICC)	95% CI	P	Inter-reliability(ICC)	95% CI	*P*
Morphological Grade	1	0.72	0.66–0.77	< 0.01	0.61	0.55–0.66	< 0.01
	2	0.78	0.73–0.82	< 0.01			
Nerve Root Sedimentation	1	0.85	0.81–0.88	< 0.01	0.75	0.72–0.79	< 0.01
	2	0.83	0.79–0.86	< 0.01			
Dural Sac Cross- Sectional Area	1	0.93	0.92–0.95	< 0.01	0.90	0.89–0.92	< 0.01
	2	0.91	0.89–0.93	< 0.01			

*ICC* Intra-class correlation coefficients test, *CI* Confidence interval

### Correlation analysis

#### Correlations between evaluation classifications

Each of the three classifications was correlated with the other two (*r* value ranged from − 0.58 to − 0.86, *P* < 0.01) (Table 3).

**Table 3:** The correlations between Dural Cross Sectional Area, Morphological Grade and Nerve Root Sedimentation Sign

	*r*	*P*
Dural Cross Sectional Area versus		
Morphological Grade	-0.86	< 0.01
Nerve Root Sedimentation Sign	-0.78	< 0.01
Morphological Grade versus		
Nerve Root Sedimentation Sign	-0.58	< 0.01

#### Correlations between evaluation classifications and preoperative symptoms

DCSA was correlated with LP (*r* =  − 0.14, *P* = 0.04), ODI (*r* =  − 0.17, *P* = 0.02), and claudication (*r* =  − 0.19, *P* = 0.01). The morphological grade was correlated with LP (*r* = 0.19, *P* = 0.01) and claudication (*r* = 0.27, *P* < 0.01). A correlation between the SedSign and ODI was observed (*r* = 0.23, *P* < 0.01). These three evaluation methods were not correlated with other parameters (*P* > 0.05). The correlations between evaluation classifications and preoperative symptoms are presented in Table 4.

**Table 4:** The correlations between severity of stenosis and pre-operative clinical parameters

	*r*	*P*
Dural Cross Sectional Area versus		
VAS of Leg Pain	-0.14	0.04
VAS of Low Back Pain	-0.01	0.91
ODI	-0.17	0.02
Claudication	-0.19	0.01
Morphological Grade versus		
VAS of Leg Pain	0.19	0.01
VAS of Low Back Pain	-0.09	0.20
ODI	0.09	0.20
Claudication	0.27	< 0.01
Nerve Root Sedimentation Sign versus		
VAS of Leg Pain	0.08	0.28
VAS of Low Back Pain	0.07	0.31
ODI	0.23	< 0.01
Claudication	0.02	0.83

*VAS* Visual Analog Scale, *ODI* Oswestry Disability Index

#### Correlations between evaluation classifications and postoperative clinical outcomes

DCSA was not correlated with any parameters (*P* > 0.05). The morphological grade was correlated with LP (*r* =  − 0.14, *P* = 0.04). A correlation between the SedSign and ODI was noted (*r* = 0.17, *P* = 0.02). The morphological grade and SedSign were not correlated with other parameters (*P* > 0.05). The detailed information is shown in Table 5.

**Table 5:** The correlations between severity of stenosis and post-operative clinical parameters

	*r*	*P*
Dural Sac Cross Sectional Area versus		
VAS of Leg Pain	-0.14	0.06
VAS of Low Back Pain	-0.06	0.44
ODI	-0.06	0.40
Claudication	-0.01	0.89
Morphological Grade versus		
VAS of Leg Pain	-0.14	0.04
VAS of Low Back Pain	-0.03	0.71
ODI	0.02	0.74
Claudication	0.01	0.91
Nerve Root Sedimentation Sign versus		
VAS of Leg Pain	0.07	0.30
VAS of Low Back Pain	0.09	0.23
ODI	0.17	0.02
Claudication	-0.11	0.14

*VAS* Visual Analog Scale, *ODI* Oswestry Disability Index

## Discussion

The present study sought to compare the reliability of DCSA, morphological grade, and SedSign for evaluating LSS on MRI and correlated each of them with preoperative symptoms and clinical outcomes at 12 months postoperatively. We found that there was a substantial to almost perfect intra- and inter-observer reliability for the three classifications. However, since only a few clinical parameters were weakly correlated with these classifications, no moderate or strong correlations were noted between each of these three methods and clinical outcomes preoperatively and 12 months postoperatively.

### Reliability

In this study, DCSA had the highest reliability, which was consistent with the results of Winklhofer S et al. [9]. In their study, they analyzed the intra and inter-reader reliability of the most published classifications for LSS, including five core imaging parameters and 14 additional parameters, and found that DCSA had the highest intra (0.89) and inter-reader reliability (0.81). The intra and inter-reader reliability for morphological grade in their study was 0.75 and 0.77, respectively; the inter-reader reliability was higher than that in our study. Our results for morphological grade were closer to those of Schizas et al. [6], who demonstrated an average intra-observer reliability of 0.77 ± 0.06 and inter-observer reliability of 0.67 ± 0.08.

The reliability of SedSign in our study was significantly lower than the intra and inter-observer reliability of 1.00 and 0.93, respectively, reported by Barz et al. [7] and were similar to those of Christy [8], who reported an intra and inter-observer reliability of 0.87–0.92 and 0.62–0.69, respectively. The following reasons may account for this difference. First, the modified SedSign (three subgroups) classification strengthens its value in guiding clinical decisions in theory; however, the increased number of subgroups may significantly increase the probability of inconsistent measurements. Second, the difference in reliability may be related to the heterogeneity of the images among the studies. Overall, these three classifications with substantial to almost perfect intra and inter-reliability demonstrated their excellent reproducibility.

### Correlations

Strong correlations were noted among the three classifications (ranging from − 0.58 to − 0.86, *P* < 0.01). The correlation between DCSA and SedSign was similar to that of a previous study [11, 14]. The correlation between morphological grade and DCSA was strong (− 0.86, *P* < 0.01), which was higher than that of a previous study [15]. Despite DCSA being a quantitative method, its significance was also based on the morphological degree of LSS severity and calculation [4]. Therefore, the strong correlation among the three classifications is not difficult to interpret.

Several physicians believe that the severity of stenosis is directly correlated to that of the clinical symptoms, and various radiological methods have been proposed for classifying LSS. Naturally, the effectiveness of a specific classification or grade of an imaging assessment should be primarily reflected in its correlation with symptoms and its value to guide treatment decisions. However, their correlations with symptoms and their prognostic values were uncertain [16–21].

Despite we found a trend that a more severe degree of stenosis (qualitative classifications) was associated with lesser DCSA, no strong correlation between each of the imaging classifications and clinical outcomes was noted. For preoperative clinical symptoms, several parameters were correlated with the three classifications in this study. DCSA was only correlated with LP, ODI, and claudication; morphological grade was correlated with LP and claudication; SedSign was correlated with ODI. Furthermore, the strength of correlations was weak. The result of DCSA was close to that of Ogikubo et al. [22] who reported that a smaller minimum cross-sectional area of the cauda equina was directly related to a shorter walking distance, suggesting more leg and back pain and lower health-related quality of life. However, they did not report the correlation coefficient for their analysis. In contrast, another study reported that both DCSA and morphological grade were not associated with baseline pain and function parameters [15]. Similarly, Weber C et al. [10] reported that morphological grades were not associated with preoperative ODI, back pain, and LP. In 2018, a retrospective study, including 522 patients, reported no correlations between SedSign and clinical data, such as VAS scores of LP and LBP, ODI, and claudication distance (*P* > 0.05) [11].

For postoperative clinical outcomes, only two parameters were correlated with the three classifications in this study. The morphological grade was correlated with LP (*r* =  − 0.14), and SedSign was correlated with ODI (*r* = 0.17). To date, the correlations between LSS severity and postoperative clinical outcomes remain debatable [10, 11, 15, 19, 20, 23, 24]. Weber C et al. [10] reported that no associations were noted between the morphological grade and surgical outcomes 1 year postoperatively. Another study highlighted that the postoperative outcome was clearly related to the degree of preoperative radiological LSS, including the morphological grade and DCSA [15]. However, the correlation strength was weak, and the highest correlation coefficient was − 0.28 [15]. Previous studies have shown inconsistent association between SedSign and surgical outcomes [11, 23].

Therefore, based on our results, it is difficult to conclude whether these three classifications are correlated with preoperative symptoms and surgical outcomes 12 months postoperatively or which one of these methods is better to correlate with preoperative symptoms and surgical outcomes. The following reasons may account for this. First, these methods, including DCSA, morphological grade, and SedSign, were designed to mainly evaluate the central spinal stenosis; therefore, they were insufficient to assess the lateral recess stenosis and foraminal stenosis [25]. Second, static imaging could not completely reflect the patient’s symptoms under a dynamic condition, such as during aggravated symptoms when walking or standing longer and alleviated symptoms when lying down. A previous study found that the relationship is stronger when the dural sac caliber is measured using standing/dynamic MRI [26]. Other reasons may include the heterogeneous and unspecific symptoms of LSS [10], asymptomatic LSS [27], comprehensive insurance that may result in an increased surgery demand and decreased surgery threshold [20], and different measurement methods adopted that may bias the results to some extent [17, 20, 28–30].

### Limitations

First, the retrospective design was a major limitation of this study, and some potential biases were difficult to eliminate. Second, the excluded rate of 10.3% in this study, including missing or inadequate clinical data or missing or poor quality of T2 axial MRI, may affect the results to some extent. Lastly, we did not analyze the stenosis at the L5/S1 level because the SedSign was included.

## Conclusion

This is the first study with large sample size to compare the reliability of DCSA, morphological grade, and SedSign for evaluating LSS on MRI and correlated each of them with preoperative symptoms and clinical outcomes at 12 months postoperatively. A substantial to almost perfect intra and inter-observer reliability for the three classifications was noted; however, they had either a weak correlation with symptoms and surgical outcomes or none at all. Based on our findings, using one of them without conducting other tests for LSS will have limited or uncertain value in making surgical decisions or evaluating the prognostic value.

## Data Availability

The datasets in this study are available from the corresponding author on reasonable request.

## Abbreviations

DCSA: Dura cross-sectional area
IQR: Interquartile range
LSS: Lumbar spinal stenosis
LBP: Low back pain
LP: Leg pain
MRI: Magnetic resonance imaging
ODI: Oswestry disability index
SedSign: Nerve root sedimentation sign
VAS: Visual analog scale
